# The Draft Genome of a Flat Peach (*Prunus persica* L. cv. ‘124 Pan’) Provides Insights into Its Good Fruit Flavor Traits

**DOI:** 10.3390/plants10030538

**Published:** 2021-03-12

**Authors:** Aidi Zhang, Hui Zhou, Xiaohan Jiang, Yuepeng Han, Xiujun Zhang

**Affiliations:** 1 Key Laboratory of Plant Germplasm Enhancement and Specialty Agriculture, Wuhan Botanical Garden, Chinese Academy of Sciences, Wuhan 430000, China; zhangaidi@wbgcas.cn (A.Z.); zhouhui@wbgcas.cn (H.Z.); jiangxiaohan16@mails.ucas.ac.cn (X.J.); 2 Center of Economic Botany, Core Botanical Gardens, Chinese Academy of Sciences, Wuhan 430074, China; 3 Key Laboratory of Genetic Improvement and Ecophysiology of Horticultural Crops, Institute of Horticulture, Anhui Academy of Agricultural Sciences, Hefei 230031, China; 4 University of Chinese Academy of Sciences, Beijing 100049, China

**Keywords:** flat peach, genome assembly, genome evolution, fruit flavor, terpene synthase genes

## Abstract

The flat peach has become more and more popular worldwide for its fruit quality with relatively low acidity, high sugar content and rich flavor. However, the draft genome assembly of flat peach is still unavailable and the genetic basis for its fruit flavor remains unclear. In this study, the draft genome of a flat peach cultivar ‘124 Pan’ was assembled by using a hybrid assembly algorithm. The final assembly resulted in a total size of 206 Mb with a N50 of 26.3 Mb containing eight chromosomes and seven scaffolds. Genome annotation revealed that a total of 25,233 protein-coding genes were predicted with comparable gene abundance among the sequenced peach species. The phylogenetic tree and divergence times inferred from 572 single copy genes of 13 plant species confirmed that *Prunus ferganensis* was the ancestor of the domesticated peach. By comparing with the genomes of *Prunus persica* (Lovell) and *Prunus ferganensis*, the expansion of genes encoding enzymes involved in terpene biosynthesis was found, which might contribute to the good fruit flavor traits of ‘124 Pan’. The flat peach draft genome assembly obtained in this study will provide a valuable genomic resource for peach improvement and molecular breeding.

## 1. Introduction

Peach (*Prunus persica* L.) is one of the most economically important fruits, providing plentiful vitamins, minerals, fiber and antioxidant compounds for healthy diets. Peach is also acknowledged as an ideal model for genetics and genomics studies of tree fruit species. Peach originated about 2.5 millions of years ago (Mya) in the southwest range of the Tibetan Plateau in China, and its cultivation and domestication in China can be traced back to 4000 years ago [1]. More than 1000 cultivars of *Prunus persica* L. (*P. persica)* were produced worldwide until now, with significant phenotypic changes in internal and external characteristics, such as fruit shape, fruit size, flavor, and flower type. However, due to selfing as well as important bottlenecks in its recent breeding history, peach has a lower level of genetic variability compared with other Prunus crops. The edible *Prunus ferganensis (P. ferganensis)*, which is native to arid regions of central Asia and featured with long unbranched leaf veins and longitudinal grooves on the pit, was a close relative of cultivated peach, and classified as a species currently [2,3,4].

The flat peach was cultivated in China two thousand years ago and introduced to Western countries from China in the seventeenth century. Featuring a saucer fruit shape, flat peach was previously supposed to be a natural mutation variety of round peach [5,6]. In early era, the flat fruit shape trait was negatively selected in most breeding programs in 70 western countries due to its effects on fruit size and yield [7]. However, compared with most round peaches, flat peaches demonstrated special germplasm characteristics for multiple unique or high quality traits [8]. Most flat peach varieties have a sweet taste, low titratable acidity (less than 0.4%), very high sugar content and rich flavor (soluble solids content of 12–14% and soluble sugars content of 9.01 and 10.69%), these high quality traits attracted more and more interest from consumers and breeders [8]. Compared with most native cultivars, the newly-bred varieties had a better quality, wider ripening period and improved fruit weight, especially richer fruit aroma. The mixture of terpene volatiles released by the peach fruits contained high levels of terpenes linalool [9]. Among these improved cultivars, ‘Sahuahongpantao’, ‘Wanshudapantao’ and ‘124 Pan’ were proved to be good flat peach cultivars [8]. Although *P. ferganensis* has a flat shape, its fruit quality is not of commercial standard in terms of fruit firmness, quality, and skin color.

As a diploid species (2*n* = 16) with a small genome (approximately 220 Mb, about twice that of Arabidopsis), peach was used as a model fruit species in comparative and functional genomics, especially Rosaceae family [10]. The peach genome released by the International Peach Genome Initiative provides a foundation for population analyses of peach [11]. In a previous study, we explored the reason for the flat fruit shape in peach and proposed that a 1.7 Mb chromosomal inversion downstream of a *P. persica* OVATE family protein 1 (PpOFP1) was responsible for the flat fruit shape in peach [12]. However, the reason why flat peach has a higher fruit quality remains unclear. To better exploit the resource of flat peach, the availability of whole-genome sequences is crucial.

To investigate the genome of flat peach, the cultivar ‘124 Pan’ was used for whole-genome sequencing and assembling. As a cross breeding variety produced by the Institute of Agricultural Science in Lixia-he Area of Jiangsu Province in 1957, ‘124 Pan’ was characterized by high fruit yield and quality, such as low acidity, high sugar content and rich flavor. Based on PacBio and Illumina reads with approximately 80-fold coverage of peach reference, we assembled a draft genome of ‘124 Pan’ by using a hybrid assembly algorithm. We compared the genome of ‘124 Pan’ with peach Lovell reference and discovered a large number of single nucleotide polymorphisms (SNPs), insertions or deletions of DNA segments (InDels), and structural variations (SVs). Through a series of comparative analysis, we confirmed that *P. ferganensis* was the ancestor of the domesticated peach. Gene family comparison revealed the expansion of terpene synthase genes (TPSs) in ‘124 Pan’, which might contribute to its good fruit flavor traits. This flat peach draft genome assembly provides an extra resource for peach improvement and comparative genomics research.

## 2. Results

### 2.1. Landscape of Genome Variations of ‘124 Pan’

**Figure 1:**
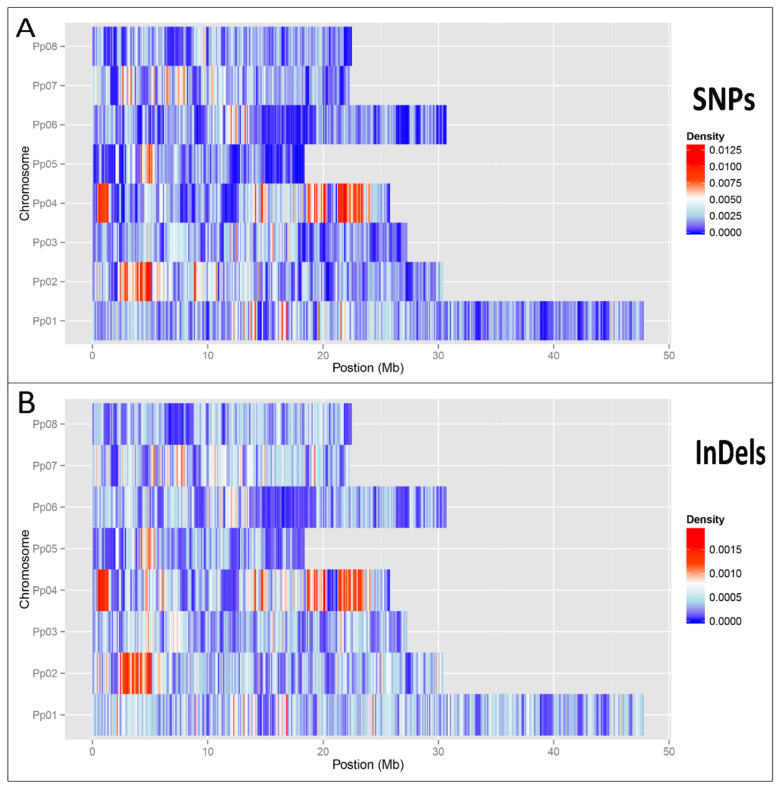
Distribution of single nucleotide polymorphisms (SNPs) and insertions or deletions of DNA segments (InDels) among different chromosomes. (**A**) Density distribution of SNPs among different chromosomes. (**B**) Density distribution of InDels among different chromosomes.

The materials used for whole-genome sequencing were obtained from young leaves of ‘124 Pan’ cultivated in Wuhan Botanical Garden of the Chinese Academy of Sciences. To detect various SVs, all the Illumina clean data were aligned to the peach reference genome v2.0 (Lovell), the mapping rate was up to 97.5%. As a result, 95,124 InDels, 533,357 SNPs, and 18,422 SVs were detected (Table 1). The localization analysis showed that 2132 Indels and 41,602 SNPs were localized in the exon region. For SNPs, the number of non-synonymous sites (22,455) was higher than that of synonymous (18,725). The proportion of transitions to transversions was 1.8. In addition, T:C, C:T, T:G, T:A and C:A substitutions were the most common SNPs. Compared with peach Lovell reference, quiet a lot SVs were detected, including 2165 insertions, 3180 deletions, 82 inversions, 3675 intra-chromosomal translocations, and 2801 inter-chromosomal translocations. Many large chromosomal inversions were located in chromosomes 1, 2, 4, 6. Distribution of InDels and SNPs among different chromosomes is shown in Figure 1, which shows that the chromosomal inversions areas have higher density of SNPs and Indels (see Figure 1 in [12]), these large chromosomal variations may affect the accurate detection of the small variations.

### 2.2. Genome Assembly and Annotation of ‘124 Pan’

A total of 93,364,840 Illumina reads and 313,793 Pacbio reads were generated with the average sequencing depth correspond to 68× and 10× respectively. The average Pacbio reads length was 11,304 bp. The detailed information of the Illumina HiSeq paired-end and PacBio Sequel reads is shown in Table 2. By using a hybrid assembly algorithm, a total assembly of 206.1 Mb consisting of 2107 contigs (N50 of 170 Kb; longest contig length 1023 Kb) was constructed. All the contigs were anchored into chromosome-level pseudomolecules based on homology to the current peach Lovell v2.0 genome. The final genome assembly amounted to 206 Mb consisting of 8 chromosomes and 7 scaffolds with a N50 length of 26.3 Mb, covering about 90.8% of the peach Lovell genome. Gene prediction was performed using a combination of homology, ab initio, and transcriptome-based approaches. A total of 25,233 protein-coding genes were annotated. The assembly statistics of ‘124 Pan’ is shown in Table 3. In addition to protein-coding genes, various noncoding RNA sequences, including 550 transfer RNAs, 263 ribosomal RNAs, 122 microRNAs, and 439 small nuclear RNAs, were identified and annotated, see Supplementary Materials Table S1. Through a combination of approaches, we annotated 39.61% of the assembly as repetitive elements, see Table S2. The completeness of gene regions assessed by BUSCO (Benchmarking Universal Single Copy Orthologs) showed that 92.2% of the green plant single-copy orthologs were complete.

### 2.3. Genome Duplication and Synteny Analysis

**Figure 2:**
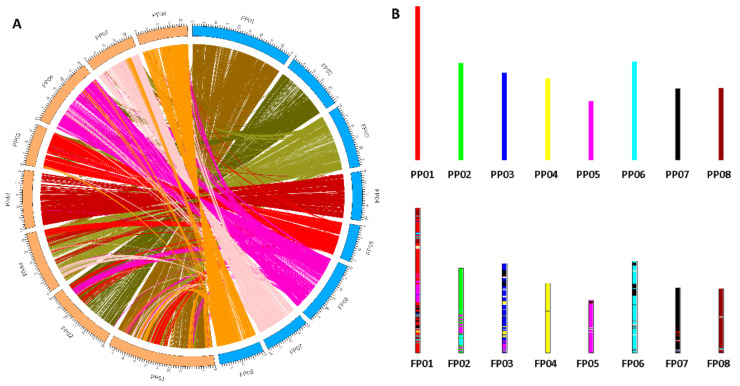
Comparative analyses of genomes between *P. persica* (Lovell) and ‘124 Pan’. (**A**) Circle plot of gene synteny. (**B**) Bar plot of chromosome fusions between *P. persica* (Lovell) and ‘124 Pan’. Different chromosomes are represented by different rainbow colors.

**Figure 3:**
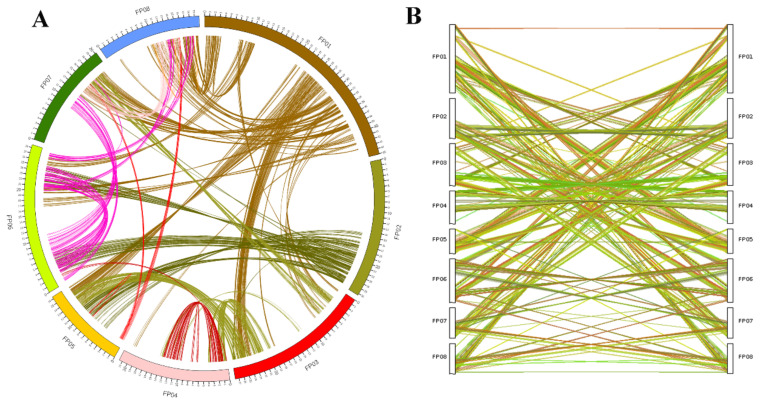
Self-collinearity of ‘124 Pan’ genome. (**A**) Circle plot of gene synteny within ‘124 Pan’ genome, different chromosomes and collinearities are distinguished by different colors. (**B**) Dual synteny of chromosome fusions within ‘124 Pan’ genome. Different collinearities are represented by different colors.

**Figure 4:**
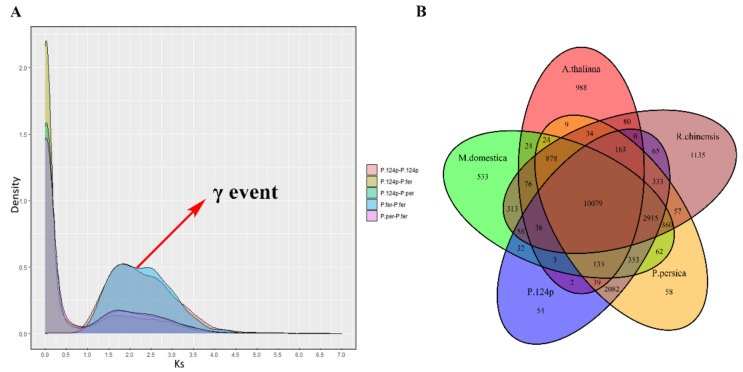
Evolutionary analyses of ‘124 Pan’ genome. (**A**) Distribution of Ks for pairs of paralogs/orthologs in/between ‘124 Pan’, *P. persica* (Lovell), and *P. ferganensis.* The eudicot hexaploidization (ancestral γ event) was indicated by red arrow. (**B**) Venn diagram of shared orthologous gene families among ‘124 Pan’ and other four genomes (*A. thaliana*, *R. chinensis*, *M. domestica*, and *P. persica* (Lovell)).

The draft genome of ‘124 Pan’ was compared with current peach Lovell v2.0 reference. The synteny analysis between them revealed that ‘124 Pan’ and peach Lovell shared basically the same chromosome structures and organization (Figure 2). Many synteny blocks were presented between different chromosomes, such as several regions of chromosome 6 were homologous to that of Lovell chromosomes 7 and 2. The self-collinearity analysis of ‘124 Pan’ detected several syntenic chromosome pairs, such as chromosomes 2 and 6, chromosomes 1 and 3, revealing the chromosomal rearrangements in the ‘124 Pan’ genome (Figure 3). Based on evidence of paleo-hexaploidization (γ event) and lineage-specific duplications in eudicots [10], these syntenic duplication blocks suggested that triplicated arrangement marks remained in the ‘124 Pan’ genome. Consistent with the previous study [11], by genome self-alignment within ‘124 Pan’ and *P. persica* by MCscanx [13], only one ancient synonymous substitution (Ks) peak around 2.0 (Figure 4A) that resulted from the triplicated arrangement (ancestral γ event) was detected, there was no recent whole-genome duplication (WGD). Divergence of ‘124 Pan’ from the ancestor of the domesticated peach *P. persica* and *P. ferganensis* was deduced based on Ks of homologous genes, as shown in Figure 4A, which shows that there is little age difference between ‘124 Pan’ and another two peaches. The peaks at a Ks mode of 0.02 for orthologs between ‘124 Pan’-*P. persica* (Lovell) and ‘124 Pan’-*P. ferganensis* genomes were essentially identical, demonstrating that these three peaches diverged from each other recently. A five-way comparison of ‘124 Pan’, three Rosaceae members (*P. persica, M. domestica* and *R. chinensis*), and *A. thaliana* using ‘OrthoFinder’ analysis yielded 20,978 gene families, of which 10,079 (48%) were shared by all five species. In this analysis, 54 gene families consisting of 178 genes unique to ‘124 Pan’ were identified, whereas 58 gene families consisting of 224 genes unique to peach Lovell were identified (Figure 4B).

### 2.4. Phylogenetic Tree Construction and Estimation of Divergence Times

**Figure 5:**
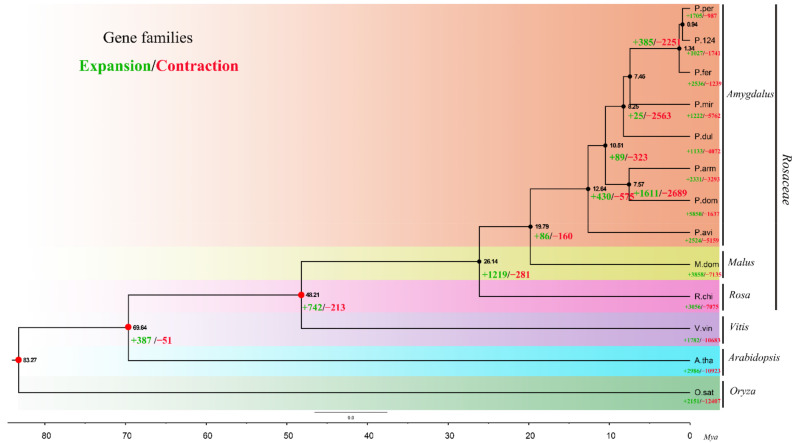
Dated phylogeny for 13 plant species with *O. sativa* as an outgroup. The timescale (Mya: Millions of years ago) is shown at the bottom. The red points in some nodes represent fossil calibration points and the black points in other nodes represent predicted fossil calibration points. Three constraints obtained from the TIMETREE website were used for time calibrations: (1) 115–308 Mya for the monocot-dicot split; (2) 98–117 Mya for the *A. thaliana*-*V. vinifera* split; (3) 107–135 Mya for the *V. vinifera*-*R. chinensis* split. The predicted divergence time was showed beside the points. The numbers in red and green font on each branch and node represent the quantity of expanded (+) or contracted (−) orthologous clusters after the corresponding speciation respectively. The 13 species were classified into six genera (*Prunoideae*, *Malus*, *Rosa*, *Vitis*, *Arabidopsis*, *Oryza*), *Prunoideae*, *Malus,* and *Rosa* belong to Rosaceae.

To investigate the orthogroups, OrthoFinder was applied to 13 species genomes including 10 members from Rosaceae, one member from Brassicaceae, one member from Vitaceae, and one member from Monocotyledon (Table 4). As a result, a total of 26,055 orthogroups were identified. Among these orthogroups, 572 were identified as putative single-copy gene families. The sequences of 572 single-copy genes in 13 species were further concatenated, aligned, and used for phylogenetic tree construction. With three selected calibration points and *O. sativa* as the outgroup, divergence time of different species was inferred using ‘MCMCtree’ (Figure 5). The topologies based on CDS and protein sequences were identical. The sister relationship and short divergence time (about 0.94 Mya) between ‘124 Pan’ and *P. persica* (Lovell) confirmed that ‘124 Pan’ is a newly diverged peach variety. In addition, *P. ferganensis* was placed as the common ancestry of ‘124 Pan’ and *P. persica* (Lovell), which were estimated to be diverged from *P. ferganensis* 1.34 Mya. The topologies also demonstrated that *P. mira* was closer to modern peach than *P. dulcis*, whereas their divergence time was estimated to be around 8.25 Mya. The age for the split of subgenera *Prunus* and *Amygdalus* was around 10.51 Mya and the divergence time from *Cerasus* was estimated to be around 12.64 Mya. These results were consistent with a previous report on the apricot genome [14] and contrary to another study about genome of wild *P. yedoensis* [15]. The latter report supposed the divergence time of subgenera *Prunus* and *Amygdalus* was around 44.0 Mya, which is higher than our estimation.

### 2.5. Gene Family Expansion and Contraction in ‘124 Pan’

Through the comparisons of the genomes among 13 species, a total of 1027 significantly (*p*-Value < 0.01) overrepresented gene families and 1741 significantly underrepresented gene families were identified in ‘124 Pan’. The dated phylogeny for 13 plant species with *O. sativa* as an outgroup is shown in Figure 5, in which the numbers of expanded/contracted orthologous for each species are denoted. To further explore the traits differentiation, the expanded and contracted orthologous in ‘124 Pan’ and *P. persica* (Lovell) were compared, and ‘124 Pan’ unique expanded and contracted orthologous were characterized. Finally, a total of 107 gene families that expanded in ‘124 Pan’ but contracted in Lovell, and 133 gene families that contracted in ‘124 Pan’ but expanded in Lovell, were identified, respectively. The annotation from Kyoto Encyclopedia of Genes and Genomes (KEGG) showed that overrepresented gene families were considerably enriched in sesquiterpenoid and triterpenoid biosynthesis, glutathione metabolism, plant-pathogen interaction, brassinosteroid biosynthesis, cyanoamino acid metabolism, and diterpenoid biosynthesis. Whereas gene families showing significant underrepresentation in ‘124 Pan’ genome were found to be involved in pathways related to secondary metabolites, phenylpropanoid biosynthesis, steroid biosynthesis, and betalain biosynthesis (Table 5).

### 2.6. Comparison of the Terpene Synthase Family among Three Peach Genomes

**Figure 6:**
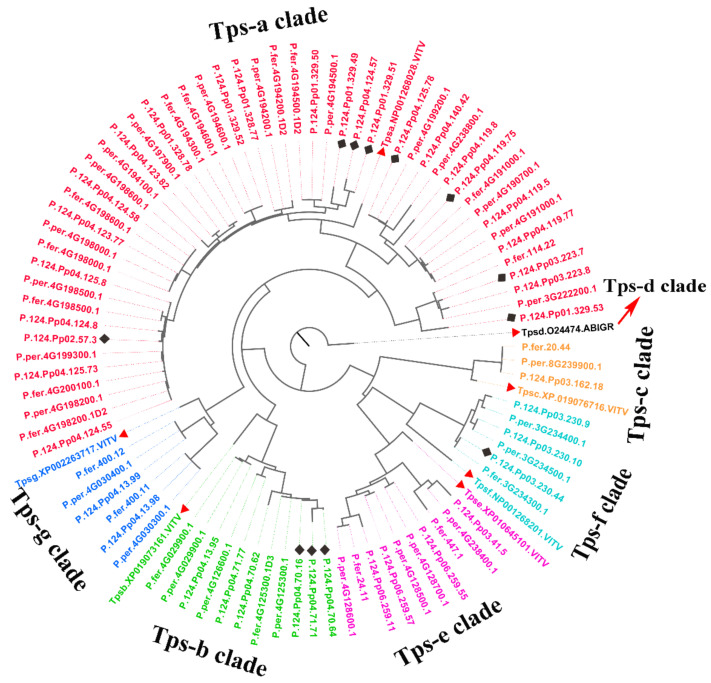
Phylogenetic tree of the TPSs family from ‘124 Pan’ and representative plants. The clades are represented by different colors. The red triangles and black diamonds on some nodes represent the representative sequences of TPS a–g and genes expanded in ‘124 pan’ respectively. The protein sequences of the seven representative TPS a–g proteins are as follows: Tpsa.NP001268028 (valencene synthase-like, *Vitis vinifera*), Tpsb.XP019073161 ((-)-alpha-terpineol synthase, *Vitis vinifera*), Tpsc.XP019076716 (ent-copalyl diphosphate synthase, *Vitis vinifera*), Tpsd_O24474 (*Myrcene synthase, Abies grandis*), Tpse.XP010645101 (ent-kaur-16-ene synthase, *Vitis vinifera*), Tpsf.NP001268201 ((E,E)-geranyllinalool synthase-like, *Vitis vinifera*), Tpsg.XP002263717 ((3S,6E)-nerolidol synthase 1, *Vitis vinifera*).

For the top enriched term of sesquiterpenoid and triterpenoid biosynthesis, most input genes belong to the TPSs family. TPSs are currently split into seven subgroups based on the phylogeny, called TPS a–g, whereas in most plants the majority of TPSs fall into one or two clades [16]. Two representative N-terminal and C-terminal domains (PF01397, PF03936) from the Pfam database were used to identify members of peach TPSs in ‘124 Pan’, *P. persica* (Lovell), and *P. ferganensis* genomes. A total of putative 19, 27, 40 TPSs were characterized in the three peach genomes, two TPSs of ‘124 Pan’ were assumed pseudogenes, whereas 38 were predicted to be functional. The higher numbers of TPSs revealed gene family expansions in ‘124 Pan’ genome. Six representative sequences of TPS-a, TPS-b, TPS-c, TPS-e, TPS-f, TPS-g in *Vitis vinifera* and one representative sequence of TPS-d in *Abies grandis* were included for alignment and phylogenetic comparison, as shown in Figure 6 and Figure S1. The phylogenetic topology revealed that all the TPSs were divided into seven known clades TPS a–g, no TPSs clustered with TPS-d that was only encoded in gymnosperms. Detailed information of TPSs is listed in Table 6. We found that most of the putative TPSs in peach genomes belonged to the TPS-a and TPS-b subfamilies, whereas ‘124 Pan’ specific expansion mainly occurred in TPS-a clade, and occasionally in TPS-b and TPS-f.

## 3. Discussion

In this study, to explore the resource of flat peach, we obtained the draft genome of one representative flat peach ‘124 Pan’ based on combination of PacBio and Illumina reads. The flat peach genome offers an opportunity to comprehensively investigate the genome variations at the resolution of nucleotide. We characterized a comprehensive catalog of structure variations, including 95,124 InDels, 533,357 SNPs, and 18,422 SVs. These genome variations constitute a major resource of genomic variation and are known to have profound consequences on phenotypic variation [17]. In plants, molecular genetic analyses have highlighted the functional importance of SVs on protein-coding and flanking noncoding regions of loci/genes linked to agriculturally important traits [17,18], the chromosomal inversion located in chromosome 6, with a size of 1.7 Mb, was proved to be responsible for the flat fruit shape in the peach [12]. The genome comparison revealed that although ‘124 Pan’ and peach Lovell shared basically the same chromosome structures and organization, there were still frequent synteny blocks presented between different chromosomes, highlighting the chromosome rearrangements events in ‘124 Pan’. Analysis of Ks distribution within ‘124 Pan’ genome revealed that there was no recent WGD except a triplicated arrangement (ancestral γ event), which is consistent with previous studies [2,11], the self-collinearity within the genome further support this point. The phylogenetic tree based on single copy genes confirmed that *P. ferganensis* was the ancestor of the domesticated peach, providing an additional evidence to support the assumption that peach domestication occurred in the region of Northwest China. The short divergence time of peach cultivars from ancestor revealed that although peach had gone through evolution by artificial and natural selection, the domesticated peach kept limited differentiation from *P. ferganensis*. Considering the same fruit shape between the cultivated flat peach and *P. ferganensis*, the flat fruit trait is more likely to have originally occurred in *P. ferganensis* and then been introduced to peach cultivars. During artificial and natural selection, the round fruit trait occurred and was selected because the round peach attracted more attention owing to its larger fruit size and higher yield, the ancient flat fruit trait was later introduced to round cultivars to breed the modern flat peach with high flavor quality.

With KEGG enrichment analysis, we found several gene family expansions in the ‘124 Pan’ genome. For the top enriched term of terpene synthesis pathway, more copies of TPSs were detected in the ‘124 Pan’ genome than that of Lovell and *P. ferganensis*. Peach TPSs were largely located in chromosome 4 that suffered drastic structural variations. For Lovell and *P. ferganensis*, TPSs were mostly located in chromosomes 4 and 3, whereas for ‘124 Pan’, additional TPSs were detected in chromosomes 1 and 2. Considering the frequent structural variation in the peach genome, we speculate that higher numbers of TPSs in flat peach are shaped by a large segment duplication and rearrangements during domestication [16]. Although Prunoideae has not experienced recent whole-genome duplication events, as observed in apple and pear [19,20], there were many translocations, large chromosomal inversions, and segment duplication regions in the genome, and the resulting chromosome rearrangements may play a key role in the speciation of Prunoideae and specialization of cultivars. For the very short evolutionary history of the modern peach, the diversity of proteins among the three peach genomes is actually small and the branch lengths in the phylogenetic tree (Figure 6) are mostly very short. Although the copy number of TPSs in flat peach is increased, the amino acid variation remains still low.

The phenomenon of varied numbers of TPSs among different peach genomes is a typical gene copy number variant (CNV). CNVs are genomic rearrangements resulting from gains or losses of DNA segments, and usually produced by transposable elements mediated nonallelic homologous recombination [21]. In plants, CNVs were mostly associated with tandem duplications and tend to occur in large families of functionally redundant genes [21]. There are demonstrated cases in which CNVs contribute to domestication and diversification traits by affecting relevant gene dosage, function(s), and/or regulation, such as rice, maize, and potato [21,22]. For TPSs, previous expression analysis showed that most duplicated copies exhibited divergent expression patterns either in tissues or transcript intensity, indicating that expression divergence significantly contributed to TPSs survival after gene expansion by duplication [16]. In addition, sequence variations within duplicated genes could generate protein diversity, hence, CNV may have enormous potential as a source of useful traits to improve cultivars [21].

Fruit flavor is a complex trait that depends on the relative amount of sugars, non-volatiles, and volatiles (terpenes, phenylalanine- and fatty acid-derived compounds) [23]. In plants, low-molecular-weight terpenes produced by TPSs are a large group of plant aromatic substances, in which monoterpenes and a few sesquiterpenes are aromatic constituents of many plants [24]. As a member of terpenes, linalool was proved to be the highest content of terpenes aromatic constituents in peach fruits [25,26]. Previous studies demonstrated that TPSs played an important role in determining the quality of horticultural food products [23,27,28]. A recent study also demonstrated the contribution of two TPSs located in chromosome 4 to carrot flavor [29]. Till now, few studies have focused on the terpenoid volatiles and peach flavors traits. Our study provided a clue to the differences of the flavor quality among peach varieties, we speculate that the striking feature of the existence of an unusually large TPSs family related to terpene biosynthesis might contribute to the fruit flavor and aroma for ‘124 Pan’. However, the distribution of TPSs among various varieties and the genetic basis underlying this trait still need further investigation in future studies. What is more, terpenoids also play numerous roles in the interactions of plants with their environment, such as attracting pollinators and defending the plant against pathogens and herbivores [24]. The plant–pathogen interaction pathway was also enriched in our study, hence, other probable relations of TPSs in peach cultivar with the improved defenses also need further investigation.

## 4. Materials and Methods

### 4.1. Plant Materials and Sequencings

Young leaves of flat peach ‘124 Pan’ from Wuhan Botanical Garden of the Chinese Academy of Sciences (Wuhan, Hubei Province) were used for whole-genome sequencing. The genomic DNA was extracted from the young leaves using a modified CATB method [30]; the DNA quality was evaluated by Qubit fluorometer and pulsed-field gel electrophoresis. The genomic DNA was sheared with a 26 G needle. Sheared DNA fragments were subjected to DNA damage repair and purified using the BluePippin automatic nucleic acid electrophoresis and fragment recovery system. A BluePippin reagent kit was used to select DNA fragments longer than 20 kb. After blunt-end ligation, adenylation and adapter ligation, the final library was sequenced using the PacBio Biosciences Sequel third-generation sequencing platform. In addition, genomic DNA of ‘124 Pan’ was also paired-end sequenced using the Illumina HiSeq X Ten sequencing system. Total RNA was extracted using Trizol reagent and RNA concentration was measured using the Thermo Scientific NanoDrop 2000. Approximately 1 μg of total RNA was used for RNA library construction. A cDNA library with insert sizes of 240 bp was prepared using the NEB Next Ultra RNA Library Prep Kit and paired-end sequenced on an Illumina platform following the manufacturer’s instructions. All the genomic Illumina paired-end sequencing data were mapped against genome assembly v2.0 of *P. persica* (peach Lovell) [10]. SNPs and InDels were identified using samtools software [31]. Sequence annotation was conducted using the ANNOVAR software [32]. BreakDancer software was used to detect structural variations, including insertion, deletion, inversion, intra-chromosomal translocation, and inter-chromosomal translocation [33].

### 4.2. Genome Assembly and Anchoring

MaSuRCA software was used to assemble the genome sequence [34]. MaSuRCA is a hybrid assembly algorithm that combines long and high-error reads with shorter but much more accurate Illumina sequencing reads. In this study, the low-error rate Illumina reads were used to build longer super-reads, which in turn were used to construct a database of all 15-mers in those reads. PacBio reads and super-reads were then aligned using the 15-mer index. Pre-mega-reads were produced and further merged to produce the final mega-reads. Finally, The mega-reads were further assembled along with the linking pairs into contigs using the CABOG assembler that was designed for long reads [35]. All the configuration parameters are default values except for setting the JF_SIZE to 2,000,000,000, LIMIT_JUMP_COVERAGE to 200. As a reference-guided contig ordering and orienting tool, RaGoo was used to anchor resulting draft genome to chromosome-scale assemblies [36]; peach Lovell genome assembly v2.0 was used as reference [10]. RNA-seq reads were assembled into transcripts using Trinity with the paired-end option and default parameters [37].

### 4.3. Gene and Repeat Annotations

*De novo* repetitive sequences in the ‘124 Pan’ genome were identified using RepeatModeler (http://www.repeatmasker.org/RepeatModeler/, accessed on 10 March 2020) based on a self-blast search. Based on the Repbase-derived RepeatMasker library and the *de novo* repetitive sequences constructed by RepeatModeler, RepeatMasker (http://www.repeatmasker.org/, accessed on 10 March 2020) was further used to search for known repetitive sequences [38]. Based on published peach Lovell proteins and *de novo* assembled transcripts of ‘124 Pan’, putative protein-coding gene structures in the ‘124 Pan’ genome were predicted based on homology and ab initio strategies using the MAKER package and Augustus [39,40]. The rRNAs were predicted using RNAmmer [41]. The tRNAs were predicted using tRNAscan-SE [42]. The noncoding RNA sequences were identified using Rfam by inner calling using Infernal [43]. The completeness of genome was assessed by performing gene annotation using the BUSCO method by searching the Embryophyta library [44].

### 4.4. Genome Synteny Analysis

Paralogs (within ‘124 Pan’ and *P. ferganensis* respectively) and orthologs (between pairs of ‘124 Pan’, *P. persica*, and *P. ferganensis*) were determined using blastp (Evalue = 1 × 10^−7^). For each gene pair, the number of synonymous substitutions per synonymous site based on the gamma-NG method was calculated using KaKs_Calculator [45]. The Ks values of all gene pairs were plotted to identify putative whole-genome duplication events. In addition, syntenic blocks between ‘124 Pan’ and *P. persica* (Lovell) were identified using the MCScanx package [13]. The collinearity of gene pairs was visualized using the Circos package [46].

### 4.5. Phylogenomic Analysis

With available genome sequences, ‘124 Pan’ and another 12 sequenced plant species, including 10 members from Rosaceae, one member from Brassicaceae, one member from Vitaceae, and one member from Monocotyledon (Table 4), were included for the phylogenetic analyses. The genome assembly of Rosaceae plants were all downloaded from GDR website (https://www.rosaceae.org/, accessed on 20 May 2020). To exclude putative fragmented genes, genes encoding protein sequences shorter than 50 aa (amino acids) were filtered out [47]. Gene redundancy caused by alternative splicing variations was removed using CD-HIT [48] (-c 0.8 aS 0.8). All filtered protein sequences of the 13 species were clustered into orthologous groups by OrthoFinder [47]. Single-copy gene families produced by OrthoFinder were used for phylogenetic tree construction. Multiple sequence alignment for protein sequences in each single-copy family was conducted using MAFFT [49]. Poorly aligned regions were further trimmed using the Gblocks [50]. The alignments of each gene family were concatenated to a super alignment matrix and then was used for phylogenetic tree reconstruction through the PROTCATJTT model in RAxML [51]. To investigate the evolutionary history of ‘124 Pan’, divergence time between 13 species was estimated using ‘MCMCtree’ with the options “independent rates” and “HKY85” model. A Markov chain Monte Carlo analysis was run for 100,000,000 generations using a burn-in of 1000 iterations [52]. Three constraints obtained from the TIMETREE website (http://timetree.org/, accessed on 2 June 2020) were used for time calibrations: (1) 115–308 Mya for the monocot-dicot split; (2) 98–117 Mya for the *A. thaliana-V. vinifera* split; (3) 107–135 Mya for the *V. vinifera-R. chinensis* split.

### 4.6. Gene Family Expansion and Contraction

For the OrthoFinder-derived orthologous gene families, gene family overrepresentation and underrepresentation were conducted using CAFÉ [53]. A birth and death process was used to model gene gain and loss across the phylogenetic tree. The distribution of family sizes was generated under this model, providing a basis for assessing the significance of the observed family size differences among taxa. For each significantly overrepresented and underrepresented gene family in ‘124 Pan’, functional information was inferred via KOBAS (http://kobas.cbi.pku.edu.cn/anno_iden.php, accessed on 20 June 2020) using KEGG Pathway database.

### 4.7. Terpene Synthase Family Analysis

Protein sequences of ‘124 Pan’, *P. persica* (Lovell), and *P. ferganensis* were scanned using pfamscan based on the HMMER suite (http://hmmer.janelia.org/, accessed on 15 August 2020). TPSs were identified by screening with the HMM profiles of the Pfam domain PF03936 (TPS, metal binding domain) and PF01397 (N-terminal TPS domain). The requirement for the presence of both domains was strict. Putative full-length TPSs (> 400 amino acids in length) were identified in the three peach species. Six representative sequences of TPS-a, TPS-b, TPS-c, TPS-e, TPS-f, TPS-g from *Vitis vinifera* from a previous study [28], and one representative sequence of TPS-d from *Abies grandis* were downloaded from UniProt database (https://sparql.uniprot.org/, accessed on 29 August 2020). Multiple protein sequence alignments of the TPS homologs were conducted using ClustalW under default settings, the neighbor-joining trees were constructed using the MEGA program (version 7.0) with 1000 bootstrap replicates [54].

## 5. Conclusions

Despite the significant advances in the reasoning for the flat shape in peach [12], the gene content that is largely responsible for the preferable fruit traits in flat peach remains relatively unexplored. ‘124 Pan’, a flat peach cultivar that is characterized by higher fruit flavor traits, was used for genome assembling and comparison. Besides a comprehensive catalog of structure variations, a flat peach genome assembly was obtained, more copies of TPSs were detected that might contribute to its flavor traits. Our study provides an extra reference for genomic variation mining and comparative studies in peach.

## Figures and Tables

**Table 1:** Summary of genomic location of SNPs, InDels and SVs in ‘124 Pan’ genome.

InDels		SNPs		SVs	
Category	Number of InDels	Category	Number of SNPs	Category	Number of SVs
Stop gain	34	Stop gain	354	\	\
Stop loss	9	Stop loss	68	\	\
Frameshift deletion	664	\	\	\	\
Frameshift insertion	563	\	\	\	\
Non-frameshift deletion	443	\	\	\	\
Non-frameshift insertion	419	\	\	\	\
\	\	Synonymous	18,725	Exonic	2188
\	\	Non-synonymous	22,455	\	\
Intronic	16,150	Intronic	65,497	Intronic	198
Splicing	114	Splicing	137	Splicing	2
Upstream	14,337	Upstream	51,641	Upstream	578
Downstream	10,536	Downstream	45,358	Downstream	427
Intergenic	49,882	Intergenic	321,567	Intergenic	2484
Insertion	45,687	\	\	Insertion	2165
Deletion	49,437	\	\	Deletion	3180
				Inversion	82
\	\	\	\	Intra-chromosomal translocation	3675
\	\	\	\	Inter-chromosomal translocation	2801
Total	95,124	Total	533,357	Total	18,422

**Table 2:** Statistics of the data used for hybrid assembly of the flat peach ‘124 Pan’.

Sequence Data Type	Number of Reads	Average Read Length (bp)	Average Sequencing Depth ^1^
Illumina HiSeq paired-end	93,364,840	150	68×
PacBio Sequel	313,793	11,304	10×

^1^ Depth is computed based on published peach Lovell v2.0 reference with genome size of 227 Mb.

**Table 3:** Summary of flat peach ‘124 Pan’ genome assembly features.

Genome Features	Contigs	Scaffolds
Total length, bp	205,882,598	206,091,798
No. of contigs/scaffolds	2107	15
Longest length, bp	1,023,978	45,096,054
Average length, bp	97,713	13,739,453
Length of N50, bp	170,447	26,342,049
Length of N90, bp	39,869	19,908,102
Guanine-cytosine content, %	37.53%	37.49%
Repeat masked, %	39.61%	39.61%
No. of genes	25,315	25,233

**Table 4:** Information of species used for phylogenetic analyses in this study.

Species	Family	Source	Genome Size (Mb)	No. of Proteins	No. of Filtered Proteins
***Oryza sativa***	Gramineae	NCBI ^1^	374.42	28,555	22,471
***Vitis vinifera***	Brassicaceae	NCBI	486.2	48,350	19,066
***Arabidopsis thaliana***	Vitaceae	NCBI	119.67	40,775	23,666
***Rosa chinensis***	Rosaceae	GDR ^2^	515.59	45,469	36,565
***Malus x domestica***	Rosaceae	GDR	703.36	45,116	30,672
***Prunus domestica***	Rosaceae	GDR	Not available	130,586	41,030
***Prunus dulcis***	Rosaceae	GDR	200.33	24,298	21,888
***Prunus avium***	Rosaceae	GDR	214.32	45,673	35,482
***Prunus armeniaca***	Rosaceae	GDR	221.9	52,096	29,658
***Prunus mira***	Rosaceae	GDR	252.39	26,958	19,157
***Prunus ferganensis***	Rosaceae	GDR	204.58	27,431	24,452
***Prunus persica***	Rosaceae	GDR	227.41	26,768	23,938
‘**124 pan**’	Rosaceae		206.1	24,513	21,874

^1^ NCBI: National Center for Biotechnology Information, https://www.ncbi.nlm.nih.gov/ (accessed on 4 February 2021). 2 GDR: Genome database for rosaceae, https://www.rosaceae.org/ (accessed on 4 February 2021).

**Table 5:** Functional annotation of the significantly overrepresented and underrepresented gene families in ‘124 pan’.

Gene Families	KEGG^1^ Terms	Input No.	Background No.	*p*-Value	Corrected *p*-Value
Overrepresented gene families	Sesquiterpenoid and triterpenoid biosynthesis	22	27	3 × 10^−9^	3.03 × 10^−7^
	Glutathione metabolism	35	103	2.82 × 10^−6^	1.42 × 10^−4^
	Plant-pathogen interaction	48	193	6.00 × 10^−5^	2.02 × 10^−3^
	Brassinosteroid biosynthesis	14	27	9.33 × 10^−5^	2.36 × 10^−3^
	Cyanoamino acid metabolism	24	99	5 × 10^−3^	0.094
	Diterpenoid biosynthesis	11	31	5.62 × 10^−3^	0.094
Underrepresented gene families	Biosynthesis of secondary metabolites	71	1126	1.5 × 10^−4^	7.54 × 10^−3^
	Phenylpropanoid biosynthesis	18	206	3.16 × 10^−5^	6.87 × 10^−4^
	Steroid biosynthesis	6	28	2.39 × 10^−4^	4.165 × 10^−3^
	Betalain biosynthesis	3	6	3.89 × 10^−3^	5.06 × 10^−2^

^1^ KEGG: Kyoto Encyclopedia of Genes and Genomes.

**Table 6:** Copy numbers of terpene synthase genes present in the peach genomes (*P. ferganensis*, *P. persica* (Lovell), and *‘124 pan’*).

TPSs Clade	Motif	P.fer	P.per	‘124 Pan’
Tps-a	DDXXD/E ^1^	11	15	24
Tps-b	DDXXD/E	2	3	6
Tps-c	DXDD	1	1	1
Tps-d	DXDD, DXXD	0	0	0
Tps-e	DDXXD/E	2	4	4
Tps-f	DDXXD	1	2	3
Tps-g	DDXXD	2	2	2
Total		19	27	40

^1^ The Asp-Asp-Xaa-Xaa-Asp/Glu (DDXXD/E) motif is important for the catalytic activity, presumably through binding to magnesium ions.

## Data Availability

The genome assembly and annotation files have been deposited under CNGB Project IDCNP0001551 (https://db.cngb.org/search/project/CNP0001551/, publicly accessible from 1 May 2021). All raw reads used in this work were deposited in NCBI Bio-Project with the accession number PRJNA588956.

## Supplementary Materials

The following are available online at https://www.mdpi.com/2223-7747/10/3/538/s1, Figure S1: Phylogenetic tree of terpene synthase family from ‘124 Pan’ and representative plants, Table S1: Statistics of noncoding RNA in ‘124 Pan’ genome, Table S2: Statistics of repeat sequences and transposable elements in in ‘124 Pan’ genome.

## Abbreviations

Ks	synonymous substitution
TPSs	terpene synthase genes
WGD	whole-genome duplication
Mya	millions of years ago
CNV	copy number variant
KEGG	Kyoto Encyclopedia of Genes and Genomes
SNPs	single nucleotide polymorphisms
InDels	insertions or deletions of DNA segments
SVs	structural variations
