# Supplementary material for: The Draft Genome of a Flat Peach (Prunus persica L. cv. ‘124 Pan’) Provides Insights into Its Good Fruit Flavor Traits
Source: Plants (Basel). 2021 Mar 12;10(3):538. doi: 10.3390/plants10030538 (PMC7998450; doi:10.3390/plants10030538)
Supplement: Supplementary file 1 [file plants-10-00538-s001.pdf]

The Draft Genome of a Flat Peach (*Prunus persica* L. cv. '124 Pan') Provides Insights into Its Good Fruit Flavor Traits

Aidi Zhang [1,2], Hui Zhou[1,2,3], Xiaohan Jiang [1,2,4], Yuepeng Han[1,2,*], Xiujun Zhang [1,2,*]

[1] Key Laboratory of Plant Germplasm Enhancement and Specialty Agriculture, Wuhan Botanical Garden, Chi-nese Academy of Sciences, Wuhan 430000, China; zhangaidi@wbgcas.cn (A.Z.); zhouhui@wbgcas.cn (H.Z.); jiangxiaohan16@mails.ucas.ac.cn (X.J.)

[2] Center of Economic Botany, Core Botanical Gardens, Chinese Academy of Sciences, Wuhan 430074, China

[3] Key laboratory of Genetic Improvement and Ecophysiology of Horticultural Crops, Institute of Horticulture, Anhui Academy of Agricultural Sciences, Hefei 230031, China

[4] University of Chinese Academy of Sciences, Beijing 100049, China

* Correspondence: yphan@wbgcas.cn (Y.H.); zhangxj@wbgcas.cn (X.Z.); Tel: +86-27-87700844 (X.Z.)

Suplementary files

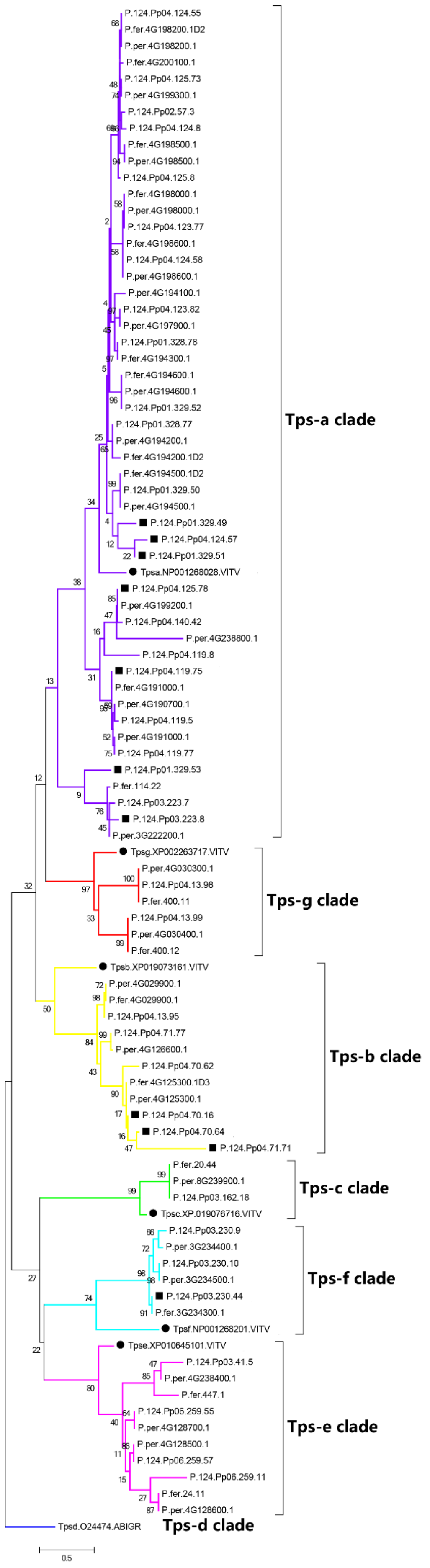
P.124.Pp04.124.55
P.fer.4G198200.1D2
P.per.4G198200.1
P.fer.4G200100.1
P.124.Pp04.125.73
P.per.4G199300.1
P.124.Pp02.57.3
P.124.Pp04.124.8
P.fer.4G198500.1
P.per.4G198500.1
P.124.Pp04.125.8
P.fer.4G198000.1
P.per.4G198000.1
P.124.Pp04.123.77
P.fer.4G198600.1
P.124.Pp04.124.58
P.per.4G198600.1
P.per.4G194100.1
P.124.Pp04.123.82
P.per.4G197900.1
P.124.Pp01.328.78
P.fer.4G194300.1
P.fer.4G194600.1
P.per.4G194600.1
P.124.Pp01.329.52
P.124.Pp01.328.77
P.per.4G194200.1
P.fer.4G194200.1D2
P.fer.4G194500.1D2
P.124.Pp01.329.50
P.per.4G194500.1
P.124.Pp01.329.49
P.124.Pp04.124.57
P.124.Pp01.329.51
Tpsa.NP001268028.VITV
P.124.Pp04.125.78
P.per.4G199200.1
P.124.Pp04.140.42
P.per.4G238800.1
P.124.Pp04.119.8
P.124.Pp04.119.75
P.fer.4G191000.1
P.per.4G190700.1
P.124.Pp04.119.5
P.per.4G191000.1
P.124.Pp04.119.77
P.124.Pp01.329.53
P.fer.114.22
P.124.Pp03.223.7
P.124.Pp03.223.8
P.per.3G222200.1
Tps-a clade
Tpsg.XP002263717.VITV
P.per.4G030300.1
P.124.Pp04.13.98
P.fer.400.11
P.124.Pp04.13.99
P.per.4G030400.1
P.fer.400.12
Tps-g clade
Tpsb.XP019073161.VITV
P.per.4G029900.1
P.fer.4G029900.1
P.124.Pp04.13.95
P.124.Pp04.71.77
P.per.4G126600.1
P.124.Pp04.70.62
P.fer.4G125300.1D3
P.per.4G125300.1
P.124.Pp04.70.16
P.124.Pp04.70.64
P.124.Pp04.71.71
Tps-b clade
P.fer.20.44
P.per.8G239900.1
P.124.Pp03.162.18
Tpsc.XP.019076716.VITV
Tps-c clade
P.124.Pp03.230.9
P.per.3G234400.1
P.124.Pp03.230.10
P.per.3G234500.1
P.124.Pp03.230.44
P.fer.3G234300.1
Tpsf.NP001268201.VITV
Tps-f clade
Tpse.XP010645101.VITV
P.124.Pp03.41.5
P.per.4G238400.1
P.fer.447.1
P.124.Pp06.259.55
P.per.4G128700.1
P.per.4G128500.1
P.124.Pp06.259.57
P.124.Pp06.259.11
P.fer.24.11
P.per.4G128600.1
Tps-e clade
Tpsd.O24474.ABIGR
Tps-d clade
0.5

Fig. S1 Phylogenetic tree of terpene synthase family from '124 Pan' and representative plants**.**

Table S1. Statistics of noncoding RNA in '124 Pan' genome

Type		Copy	Average length (bp)	Total length (bp)
tRNA		530	75	39,486
rRNA	18S	8	910	7,278
	28S	14	538	8,604
	5.8S	4	210	841
	5S	237	110	26,222
snRNA	CD-box	293	102	29,932
	HACA-box	37	129	4,790
	splicing	109	150	16,367
miRNA		122	123	14,948

Table S2. . Statistics of repeat sequences and transposable elements in in '124 Pan' genome

**Type**	**Numbers**	**Repeat size (bp)**	**Percent of genome (%)**
**SINEs**	3,401	312,468	0.15%
**LINEs**	3,220	1,353,121	0.66%
**LTR**	40,239	29,712,668	14.43
**DNA**	45,735	21,308,959	10.35
**Unclassified**	84,784	24,841,394	12.07
**Simple repeats**	70,260	2,693,097	1.31
**Satellites**	1,578	847,847	847847
**Low complexity**	12,986	629,185	0.31
